# Expression of EPHRIN-A1, SCINDERIN and MHC class I molecules in head and neck cancers and relationship with the prognostic value of intratumoral CD8^+^ T cells

**DOI:** 10.1186/1471-2407-13-592

**Published:** 2013-12-11

**Authors:** Meriem Hasmim, Cécile Badoual, Philippe Vielh, Françoise Drusch, Virginie Marty, Agnès Laplanche, Mariana de Oliveira Diniz, Hélène Roussel, Eléonore De Guillebon, Stéphane Oudard, Stéphane Hans, Eric Tartour, Salem Chouaib

**Affiliations:** 1 U753-INSERM, Institut Gustave Roussy, 114 rue Edouard Vaillant, 94800 Villejuif, France; 2 INSERM U970 PARCC, Universite Paris Descartes, Sorbonne Paris Cité, Faculté de médecine Descartes, Paris, France; 3 Service d’Anatomie Pathologique, Hôpital Européen Georges Pompidou, Paris, France; 4 Département de Biopathologie et Centre de Ressources Biologiques, Institut Gustave Roussy, 114 rue Edouard Vaillant, 94800 Villejuif, France; 5 Laboratoire de Recherche Translationnelle, Module d’HistoCytoPathologie, Institut Gustave Roussy, 114 rue Edouard Vaillant, 94800 Villejuif, France; 6 Service de Biostatistique et d'Épidémiologie, XU521 INSERM, Institut Gustave Roussy, 114 rue Edouard Vaillant, 94800 Villejuif, France; 7 Service d’Oncologie Médicale, Hôpital Européen Georges Pompidou, Université René Descartes, Sorbonne Paris Cité, Faculté de médecine, Paris, France; 8 Service d’ORL, Hôpital Européen Georges Pompidou, Université René Descartes, Sorbonne Paris Cité, Faculté de médecine, Paris, France

## Abstract

**Background:**

Our group has previously shown that EPHRIN-A1 and SCINDERIN expression by tumor cells rendered them resistant to cytotoxic T lymphocyte-mediated lysis. Whereas the prognostic value of EPHRIN-A1 expression in cancer has already been studied, the role of SCINDERIN presence remains to be established. In the present work, we investigated the prognosis value of EPHRIN-A1 and SCINDERIN expression in head and neck carcinomas. In addition, we monitored the HLA-class I expression by tumor cells and the presence of tumor-infiltrating CD8^+^ T cells to evaluate a putative correlation between these factors and the survival prognosis by themselves or related to EPHRIN-A1 and SCINDERIN expression.

**Methods:**

Tumor tissue sections of 83 patients with head and neck cancer were assessed by immunohistochemistry for the expression of EPHRIN-A1, SCINDERIN, HLA class I molecules and the presence of CD8^+^ T cells.

**Results:**

No significant prognosis value could be attributed to these factors independently, despite a tendency of association between EPHRIN-A1 and a worse clinical outcome. No prognostic value could be observed when CD8^+^ T cell tumor infiltration was analyzed combined with EPHRIN-A1, SCINDERIN or HLA class I expression.

**Conclusion:**

These results highlight that molecules involved in cancer cell resistance to cytotoxic T lymphocytes by themselves are not a sufficient criteria for prognosis determination in cancer patients. Other intrinsic or tumor microenvironmental features should be considered in prognostic evaluation.

## Background

Head and neck cancer accounts for more than 550,000 cases annually worldwide and is the seventh most common cause of cancer-induced mortality [1,2]. Immune or tumor related biomarkers such as serum cytokines, soluble cytokine receptors, metalloproteinases, as well as EGF-R expression and P53 mutations in tumor cells have been identified as useful molecules for prognostic evaluation [3-6]. However, because of their lack of specificity or sensitivity and the need to reproduce these results in multicentric studies, they have not been used in the clinical practice so far.

T cell infiltration is emerging as novel type of cancer biomarker based on the host immune response. Tumor-infiltrating cytotoxic CD8^+^ T cells, in particular, are capable of mediating directly the death of tumor cells via the release of cytokines (IFNγ, TNF), cytotoxic factors (perforin, granzymes) or the engagement of ligand-receptor interactions from the TNF family (TRAIL-TRAIL-R, FasL-Fas, TNF-TNF-R) [7,8]. CD8^+^ T cells may also inhibit or prevent cancer progression by favoring the intratumoral recruitment of immune effectors including neutrophils, macrophages and NK cells, promoting the amplification of the *in situ* anti-tumor immune responses [9-11].

A positive correlation between the patient survival and the presence of a CD8^+^ T cell tumor infiltrate has been reported [12-15]. These observations are further supported by preclinical models that showed that the CD8^+^ T cell concentration determines their cytolytic function [16] and that tumor regression was mediated by CD8^+^ T cells in mice [17]. On the other hand, high densities of CD8^+^ T cells have also been associated with tumor progression. In this regard, several studies on renal cell carcinomas and hematological malignancies have reported that high CD8^+^ T cell concentrations correlated with bad prognosis [18-21].

Previously, our group has shown that CD8^+^ T infiltration in head and neck squamous cell carcinomas has no prognostic value [22]. Several factors could be associated with the lack of prognostic significance associated with intratumoral CD8^+^ lymphocytes: absence or loss of HLA-class I molecules on tumor cells, resistance of tumor cells to immune attacks [23-28] and an anergic state of CD8^+^ T cells mediated by the expression of negative co-stimulatory molecules such as PD-1 [29,30].

EPH receptor tyrosine kinases and their EPHRIN ligands constitute a large cell communication system with the ability of generating bidirectional signaling: forward signals in EPH-receptor expressing cells and reverse signals in EPHRIN ligand-expressing cells [31]. These signals can result in cytoskeleton reorganization, cell adhesion or cell separation. Paradoxical observations regarding expression of EPH receptors and EPHRINS in cancer have been reported. Indeed, the up and down-modulation of several EPH receptors and EPHRINS have been described in different types of cancers compared to their healthy tissues of origin [31]. The upregulation of EPHRIN-A1 in hepatocellular carcinoma promoted the proliferation and the expression of genes associated with proliferation and invasion [32]. The expression of EPHRIN-A1 and its major receptor EPH-A2 were both augmented and found to be significantly associated with worse prognosis in oesophageal squamous cell carcinoma where they correlated with shorter survival [33]. Greater expression of EPHRIN-A1 and EPH-A2 were also found in adenoid cystic carcinoma of the salivary gland and correlated with the tumor stage [34]. However, unbalanced expression of EPHRIN-A1 and EPH-A2 was reported in several cancers such as glioblastoma where in the absence of EPHRIN-A1, EPH-A2 becomes a substrate for Akt, promoting cell migration and invasion [35]. The oncogenic properties of EPH and EPHRIN signaling have also been analyzed. In this respect, EPH-dependent signals have been reported to have tumor suppressive functions [36-38].

SCINDERIN is a member of the geslolin family of actin-severing proteins which regulate the actin cytoskeleton by severing pre-existing actin filaments, capping filaments ends and nucleating actin assembly from monomers in a calcium-dependent manner [39,40]. SCINDERIN has been reported to play a role in exocytosis by mediating disruption of F-actin cortical network, whereas the other members of the gelsolin family, such as gelsolin, contribute to cell motility. An anti-tumor role for SCINDERIN has been described based on its capacity to induce differentiation and apoptosis in megakaryoblastic leukemia cells that overexpress SCINDERIN [41]. In contrast, increased expression of SCINDERIN has recently been related to resistance of bladder tumor cells to cisplatin via inhibition of mitochondria-mediated apoptosis [42].

We have previously reported that increased EPHRIN-A1 and SCINDERIN expression in tumor cells conferred resistance to CD8^+^ T cell-mediated lysis by a mechanism linked to actin cytoskeleton remodeling [26,43]. While previous studies have shown increased expression of EPHRIN-A1 in head and neck cancers [44], the expression of SCINDERIN remains unestablished in these tumors. Our previous observations in head and neck cancer indicated no prognostic significance for CD8^+^ T cell infiltration [22,30]. Considering this, the present study was designed to address whether the expression of proteins involved in the resistance to cytotoxic CD8^+^ T cells such as EPHRIN-A1 and/or SCINDERIN or the downmodulation of HLA-class I molecules, could explain the absence of clinical impact of intratumoral CD8^+^ T cell infiltration in head and neck cancer.

## Methods

### Ethic statement

This study was conducted in accordance with the french laws and was approved by the local ethic committee (Comité de Protection des Personnes "Ile de France II", n°ID RCB 2007-A01128-45). Patients provided their written informed consent (approved by the ethic committee) to participate in this study.

### Patients

83 patients with untreated and histologically diagnosed head and neck squamous cell carcinoma were enrolled for EPHRIN-A1 and SCINDERIN expression analysis from January 2004 to December 2011. Each patient disease stage was classified according to the 7^th^ edition of the International Union Against Cancer/American Joint Committee on Cancer system for head and neck cancer. Treatments consisted of chemo-radiotherapy without surgery (organ preservation) or surgery combined or not with chemo-radiotherapy. Patients were matched for various parameters (gender, primary tumor site, tumor staging, lymph node involvement, presence of metastasis, HPV status and treatment modalities; Table 1).

**Table 1:** Patient characteristics

**Category**	**Subcategory**	**Results**
***Sex***	Male	75
	Female	8
***Age***	<50 y	7
	50-65 y	55
	≥66 y	21
***Primary tumor site***	Oral cavity	10
	Oropharynx	31
	Hypopharynx	16
	Larynx	26
***Tumor (T) staging***	T1	6
	T2	19
	T3	26
	T4	32
***Lymph node involvment***	N0	22
	N1	11
	N2	41
	N3	8
	N4	1
***Metastasis***	M0	76
	M1	7
***Treatment***	Surgery	8
	Chemoradiotherapy	35
	Surgery + chemoradiotherapy	39
	None	1
***HPV status***	Positive	14
	Negative	59
	Not determined	10

### Immunohistochemical staining

Frozen sections (4 μm thick) of tumors were fixed in formol 4%, transferred in Citrate Buffer pH = 6 (MM France, T/T0050) in decloaking chamber for 20 min, blocked with blocking buffer (Dako, Glostrup, Denmark; X0909) and stained either with anti-SCINDERIN (Sigma-aldrich, St. Louis, MO, USA; 2.75 μg/ml), anti-EphrinA1 (Santa-Cruz biotechnologies, Santa Cruz, CA, USA; 0.7 μg/ml) or anti-HLA class I (anti-β2 microglobuline antibody produced in our laboratory, 0.05 μg/ml) antibodies.

Tissue sections were further treated with peroxydase-conjugated secondary antibodies (DAKO, Glostrup, Denmark; EnVision K4003) and counterstained with Mayer’s hemalum for better nuclei visualisation.

HPV status (Table 1) was estimated by immunochemistry using prediluted anti- p16 INK4a CINtec® antibody (Roche) for 73 patients out of 83. Samples from the remaining 10 patients were not available due to the small size of the biopsies. The staining was performed using the BenchMark Clyde ULTRA Ventana Roche apparatus.

**Figure 1:**
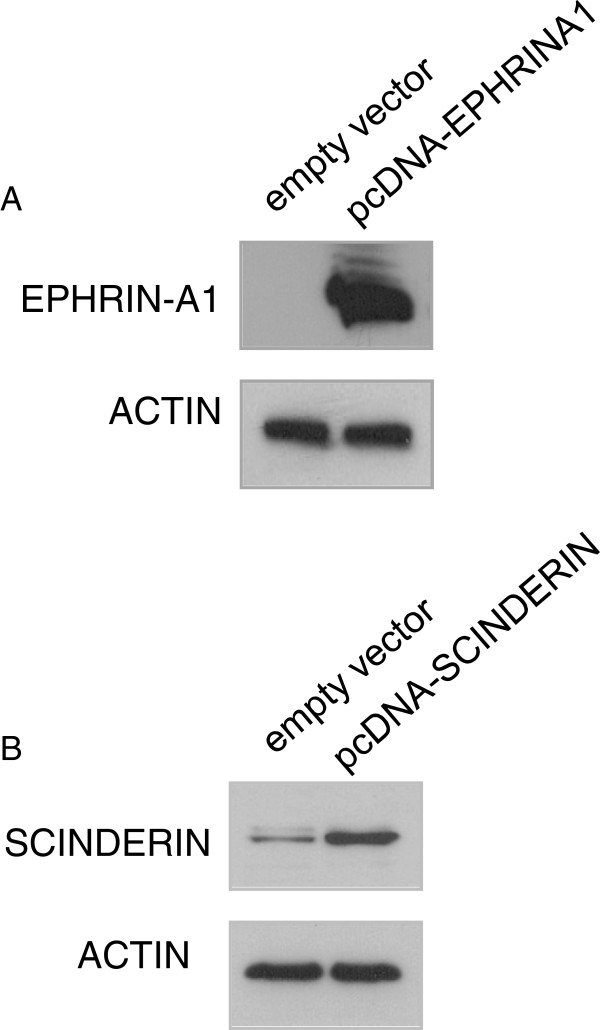
**Western-blot analysis of anti-EPHRIN-A1 and anti-SCINDERIN antibody specificity.** Non-small cell lung carcinoma cells (IGR-Heu) were transfected with empty vector or plasmids encoding either EPHRIN-A1 or SCINDERIN. Antibody specificity was further assessed by western-blot analysis of protein cell extracts.

The specificity of anti-EPHRIN-A1 and anti-SCINDERIN antibodies was assessed by Western-blot on the tumor cells (IGR-Heu cells issued from a non-small cell lung carcinoma biopsy) transfected with empty vector or plasmids encoding either EPHRIN-A1 (Figure 1A) or SCINDERIN (Figure 1B). Transfections were performed using Lipofectamine® 2000 reagent (Invitrogen).

### Immunofluorescence staining

Tissue samples obtained before any treatment at initial endoscopy or surgery were immediately frozen and stored at −80°C. Frozen specimens were sectioned at 4 μm with a cryostat, placed on slides, air dried and fixed for 10 min. Before incubation with anti-CD8 (Abcam; ab4055; 1 μg/ml) antibody, the slides were treated with avidin/biotin blocker (Vector Laboratories, Burlingame, CA) and Fc receptor was blocked with human serum (5%). Isotype-matched antibodies were used as negative controls. Fluorescent images of mounted sections were analyzed with an epifluorescent microscope (DMR, Leica Microsystems, Wetzlar, Germany).

### Scoring procedure

**Figure 2:**
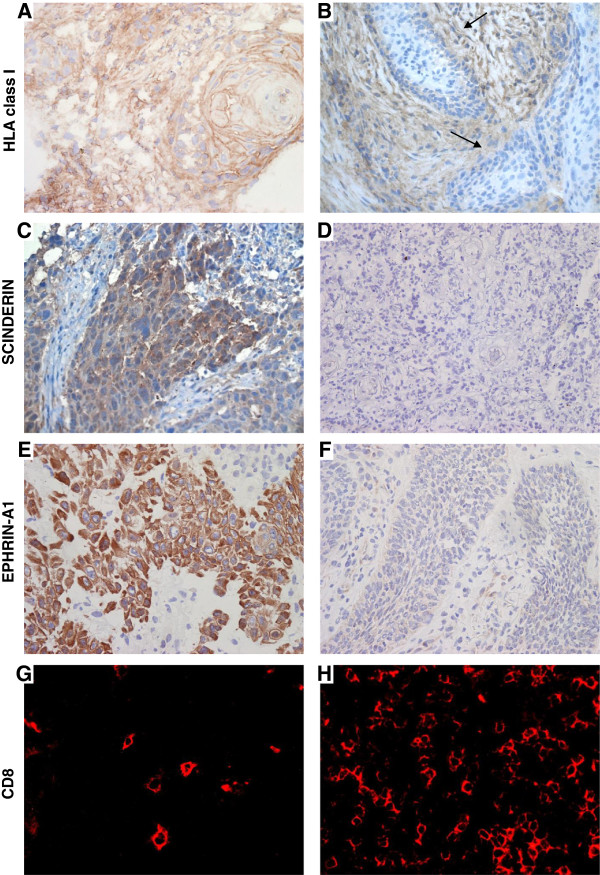
**Immunochemical and immunofluorescent staining with the anti-W6-32, SCINDERIN, EPHRIN-A1 and CD8 antibodies in squamous cell carcinoma.** Tissue sections derived from biopsies of head and neck cancers were stained with antibodies to human HLA class I, SCINDERIN, EPHRIN-A1 and CD8. **A** (positive), **B** (negative -arrows) HLA class I stainings; **C** (positive), **D** (negative) SCINDERIN stainings; **E** (positive), **F** (negative) EPHRIN-A1 stainings; **G** (low), **H** (high) CD8^+^ T cell infiltrations.

A semi-quantitative grading system was used to quantify the tumor infiltrating CD8^+^ T cell subpopulations, EPHRIN-A1 and HLA-class I expression by tumor cells. The staining was scored as 0 (less than 10% positive cells), 1 (10-20% positive cells), 2 (21-50% positive cells), 3 (more than 50% positive cells) based on reference staining slides using a high magnification (x400). As previously reported, the cut-off for high and low expression group was arbitrarily set up as > or ≤ 20% positively stained cells respectively [30]. For SCINDERIN expression, only the presence or the absence of its expression by tumor cells was recorded. Examples of staining are shown in Figure 2. Level of expression for each marker is summarized in Table 2.

**Table 2:** Number of patient for each marker expression

	**0**	**1**	**2**	**3**
**CD8**	6	38	21	18
**HLA-class I**	32	38	12	1
**EPHRIN-A1**	33	12	6	32
**SCINDERIN**	74	9		

Except for SCINDERIN, a marker expression of 0 to 1 is considered as low and of 2 to 3 as high.

The mean of positively stained cells in at least five fields using a 40x objective was selected for each sample. Two authors (C.B. and E.T.) blinded for clinical data, independently scored the slides.

### Statistical analyses

The relationship between the different staining was analyzed by the m2 test or the Fisher’s exact test as necessary. Survival variables were estimated using the Kaplan-Meier method and compared by the log-rank test. Multivariate analysis using the Cox proportional hazard model determined the influence of each variable, when adjusted to others, on locoregional control (relapse free survival) and overall survival. The level of significance was set at P V 0.05. Overall survival was defined as the time from initial diagnosis until death or until last follow-up (right censored data). Overall survival took all deaths into account. Locoregional control was calculated from the end of treatment and was defined as the absence of disease or either persistent or recurrent disease at the primary site or in the cervical lymph nodes. Patients with persistent disease at the end of treatment were considered to have experienced failure at time zero. Patients with no signs of relapse were censored at the time of last follow-up or death.

## Results

### Prognosis value of treatments in head and neck cancers

**Figure 3:**
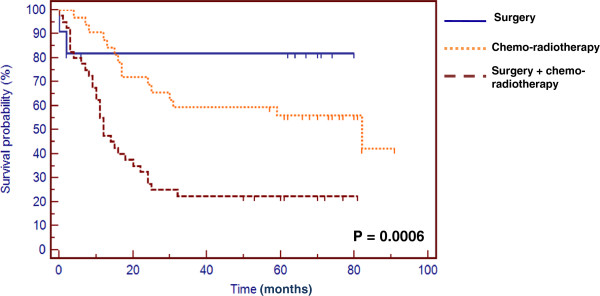
**Overall survival in head and neck carcinoma treated by surgery, chemo-radiotherapy or surgery combined with chemo-radiotherapy.** Overall survival of 83 head and neck carcinoma patients based on the type of treatments.

Patients who were treated by surgery alone or combined with chemo-radiotherapy had a better overall survival than patients treated by chemo-radiotherapy alone, as shown in Figure 3. In this work, we did not focus on the prognostic value of the clinical parameters (sex, age, primary tumor site, TNM staging) listed in Table 1 as it was not the aim of the current study. We and others have already addressed their prognostic value in head and neck cancers and found that the TNM classification was correlated with overall survival, whereas sex, age and primary tumor site had no impact on the clinical outcome [4,45].

### CD8^+^ T cell infiltration was not associated with an improved prognosis in head and neck cancer patients

**Figure 4:**
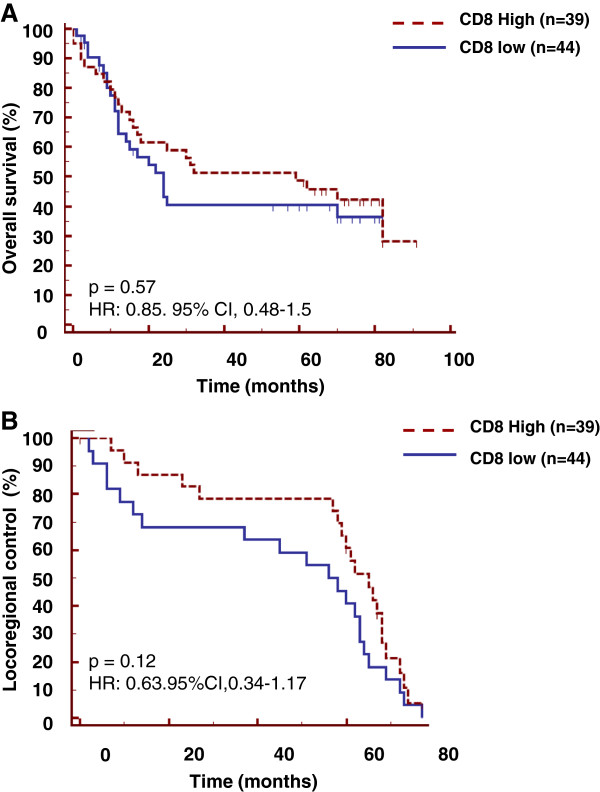
**No prognostic value of intratumoral CD8**^**+ **^**T cells in head and neck cancer.** Tissue sections derived from biopsies of head and neck cancers were stained with the anti-CD8 antibody by simple immunofluorescence analysis. For overall survival analysis **(A)** and disease free survival analysis **(B)**, high and low levels of these populations were defined as low (≤ 20% positively stained tumor cells) versus high (> 20% positively stained cells).

In our series of 83 patients, those with high infiltration of CD8^+^ T cells (n = 39), defined by more than 20% of infiltrating cells (see Methods), had a median overall survival time of 59 months. Patients with low infiltration of CD8^+^ T cells (n = 44) presented a median overall survival time of 24 months (Figure 4A), however this difference did not reach statistical significance (HR: 0.85. 95% CI, 0.48-1.5; p = 0.57). In accordance with these results, the difference in CD8^+^ T cell infiltration in head and neck cancer patients did not confer significant advantage on locoregional control (relapse free survival) (p = 0.12) (Figure 4B). Another cut-off selected to discriminate low or high CD8^+^ T cell infiltration did not change the absence of correlation between CD8^+^ T cell infiltration and prognosis (data not shown).

We next attempted to investigate some tumoral features known to impact CD8^+^ T cell activity.

### The non prognostic value associated with intratumoral CD8^+^ T cells does not correlate with a decrease in tumor MHC class I molecules

**Figure 5:**
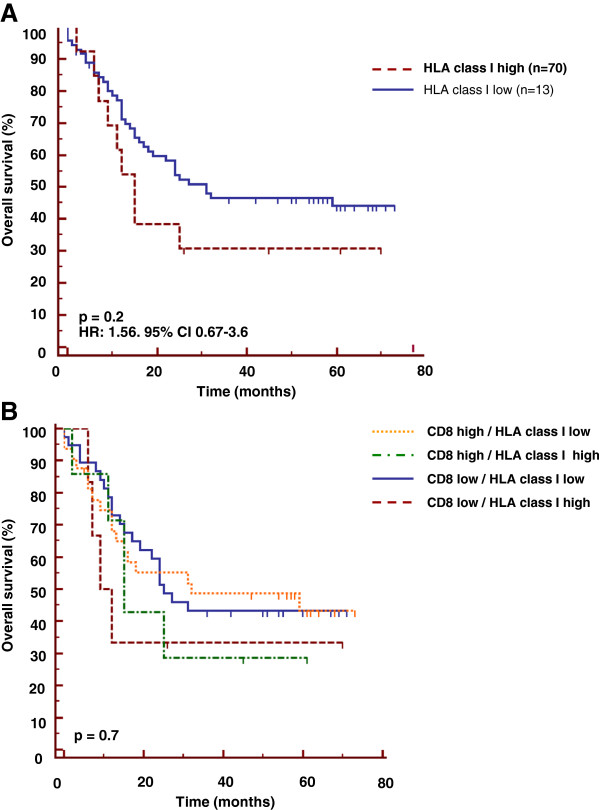
**Expression of HLA class I by tumor cells is not associated with prognosis and does not improve the prognostic value of intratumoral CD8**^**+**^**T cells. A)** Tissue sections derived from biopsies of head and neck cancers were stained with the anti-HLA class I antibody by simple immunochemical analysis. For overall survival analysis, high and low levels of these populations were defined as low (0, 1) versus high (2, 3). **B)** CD8 and HLA-class I stainings were measured in biopsies derived from head and neck cancer patients. The combination of these two stainings and the overall survival are represented. For overall survival analysis, the high and low groups for each population were combined.

Since the anti-tumor activity of CD8^+^ T cells requires the expression of HLA-class I molecules by tumor cells, we assessed its expression in head and neck cancers. Using various cut-off we did not find any correlation between the presence of HLA-Class I molecules detected by an anti-β2 microglobulin mAb and the CD8^+^ T cell infiltration (data not shown). We found that 32 out of 83 patients did not express HLA-class I molecules and 38 patients express HLA class I molecules in less than 20% of cells. These two groups of patients were gathered in the low HLA class I expressing group. Thirteen tumors from head and neck cancer patients expressed high levels of HLA-class I molecules. Surprisingly, patients whose tumors expressed low levels of HLA-class I molecules exhibited a better prognosis as assessed by median overall survival (31 months), than patients expressing high levels of HLA-class I molecules in their tumors (15 months) (Figure 5A). However, this difference was not statistically significant (HR: 1.56. 95% CI 0.67-3.6; p = 0.2).

When we combined the analyses of low and high intratumoral CD8^+^ T cell infiltration and the low and high HLA-class I expression groups, it did not improve the overall survival (Figure 5B) (p = 0.7). Unexpectedly, patients with high CD8^+^ T cell infiltration and low HLA-class I expression presented a poor outcome, as the overall survival analysis of this group was 15 months compared to the 25 months overall survival for the group of patients with low CD8^+^ T cell infiltration and low expression of HLA-class I molecules (Figure 5B).

### Expression of EPHRIN-A1 by tumor cells tends to be associated with worse survival independently of CD8^+^ T cell infiltration

Since we previously demonstrated that the expression of EPHRIN-A1 and SCINDERIN by tumor cells resulted in their resistance to the lysis by anti-tumoral CD8^+^ T cells [26], we analyzed the expression of EPHRIN-A1 and SCINDERIN in head and neck cancer patients and combined the study of these cytoskeletal biomarkers with CD8^+^ T cell infiltration.

**Figure 6:**
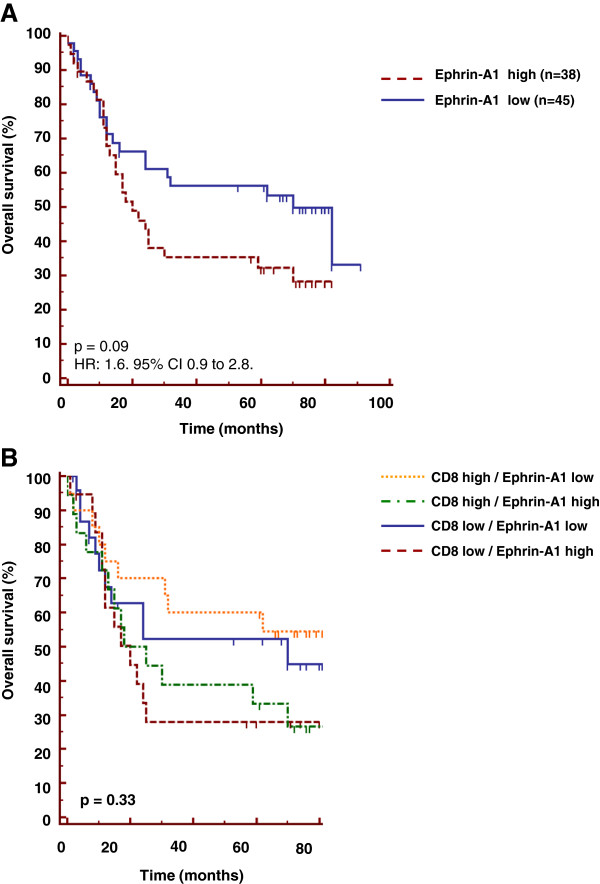
**Expression of EPHRIN-A1 tends to be associated with worse survival but does not explain the absence of prognostic value of intratumoral CD8**^**+**^**T cells. Intratumoral CD8**^**+**^**T cells in the absence of EPHRIN-A1 are not associated with a better prognosis. (A)** Tissues derived from biopsies of head and neck cancers were stained with anti-EPHRIN-A1 antibody by simple immunochemical analysis. For overall survival analysis, high and low levels of this population were defined as low (≤ 20% of positive staining cells) versus high (> 20% positive staining cells). **(B)** CD8 and EPHRIN-A1 stainings were measured in biopsies derived from head and neck cancer patients. The combination of these two stainings and the overall survival are represented. For overall survival analysis, the high and low group for each population were combined.

Using the same cut-off (> 20% positive cells) to discriminate high (n = 38) and low (n = 45) EPHRIN-A1 expression, we showed that head and neck cancer patients with high EPHRIN-A1 expression have a reduced median survival (20 months) compared to patients with low expression of EPHRIN-A1 (70 months). However, this tendency did not reach statistical significance (HR: 1.6. 95% CI 0.9 to 2.8. p = 0.09) (Figure 6A). When the CD8^+^ high and low groups were sub-classified depending on the expression of EPHRIN-A1, we found, as expected, that patients with high CD8^+^ T cell infiltration combined to those with low EPHRIN-A1 expression have a better median overall survival (82 months), than patients with low CD8^+^ T cell infiltration and high EPHRIN-A1 expression (20 months), but this difference did not reach statistical significance (p = 0.33) (Figure 6B).

### SCINDERIN expression does not correlate with prognosis in head and neck cancer and does not improve the prognostic value of the CD8 biomarker after its combination

**Figure 7:**
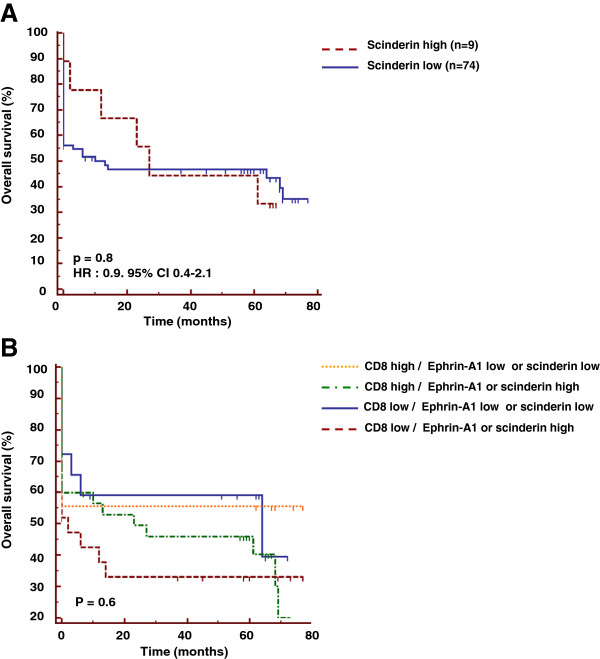
**SCINDERIN expression did not correlate with prognosis in head and neck cancer and did not explain the absence of prognostic value of intratumoral CD8**^**+**^**T cells. (A)** Tissues derived from biopsies of head and neck cancers were stained with the anti-SCINDERIN antibody by simple immunochemical analysis. For overall survival analysis, the low and high groups were defined depending on the absence or the presence of tumor SCINDERIN expression. **(B)** CD8 and SCINDERIN stainings were measured in biopsies derived from head and neck cancer patients. The combination of these two stainings and the overall survival are represented. For overall survival analysis, the high and low group for each population were combined.

SCINDERIN is weakly expressed by head and neck tumors, as only 9 out of 83 patients expressed this biomarker. Patients expressing SCINDERIN have no survival advantage compared to patients with no expression of this cytoskeletal biomarker (p = 0.8) (Figure 7A).

Due to the low number of patients expressing SCINDERIN, we combined the EPHRIN-A1 and SCINDERIN expression to address whether the presence of either SCINDERIN or EPHRIN-A1, possible surrogate markers of resistance of tumor cells to lysis mediated by CD8^+^ T cells, could influence the impact of CD8^+^ T cell on the clinical outcome of patients. Data shown in Figure 7B indicate that patients with high CD8^+^ T cell infiltration and lacking EPHRIN-A1 and SCINDERIN did not exhibit a better prognosis than the other groups of patients (Figure 7B) (p = 0.6).

When we combined all the possible mechanisms of tumor resistance to CD8^+^ T cells (HLA class I loss, EPHRIN-A1 and SCINDERIN expression), patients with high CD8^+^ T cells infiltration expressing HLA-class I molecules on tumor cells and with low tumor expression of EPHRIN-A1 and SCINDERIN did not exhibit a better prognosis than the other groups of patients (data not shown).

## Discussion

Consistent with previous data reported by our group [22,30], we found in the present study that the levels of intratumoral CD8^+^ T cell infiltration did not correlate with overall survival or locoregional control in head and neck cancer patients. This is in agreement with other reports indicating an absence of significant statistical correlation between intratumoral CD8^+^ or CD3^+^ T cell infiltration and the clinical outcome in head and neck cancer patients [46-48]. Since partial or complete loss of HLA-class I expression have been reported in head and neck tumors [49,50], we examined whether this could explain the weak impact of intratumoral CD8^+^ T cell infiltration on the clinical outcome. We found that head and neck cancer patients had a complete loss or a weak expression of HLA-class I molecules in 38.5% and 45.7% of the cases, respectively. Although it did not reach statistical significance, patients with high CD8^+^ T cell infiltration combined with low HLA class I expression exhibited a better prognosis than patients in which CD8^+^ T cell presence is associated with high HLA class I expression. This paradoxical result parallels the negative correlation between HLA expression and good clinical outcome for HPV-positive head and neck cancer, recently reported by Nasman’s group [51]. Patients with high CD8^+^ T cell infiltration and low HLA-class I expression may have experienced HLA class I molecules immunoediting, as a consequence of immune selection after the induction of an anti-tumor immune response. The absence of HLA class I molecules may represent a surrogate marker of an anti-tumor immune response and could therefore be associated with a good clinical outcome. Furthermore, low HLA class I expression could also be related to a natural killer cell immune response directed against the tumor cells.

Our group showed that SCINDERIN and EPHRIN-A1 are directly responsible for conferring resistance to tumor cells from CD8^+^ T cell attack [26]. Although a tendency between high expression of EPHRIN-A1 and overall survival was observed (p = 0.09), the expression of this cytoskeletal biomarker did not significantly correlate with the clinical outcome in patients with head and neck cancers (Figure 5 and Figure 6). A correlation between EPHRIN-A1 expression and the overall survival in head and neck squamous cell carcinomas has been previously reported [44].

The present study indicated that patients with high CD8^+^ T cell infiltration have a better prognosis, once associated with low EPHRIN-A1 expression, but this correlation did not reach statistical significance. The stratification of patients with CD8^+^T cell infiltration related to SCINDERIN expression or the combined EPHRIN-A1, SCINDERIN and HLA-class I expression did not improve the prognostic value of CD8^+^ T cell infiltration.

Different studies reported an association between intratumoral CD8^+^ T cell infiltration and a good prognosis in subgroups of head and neck cancer patients defined by the presence of HPV, their low clinical aggressiveness or their location [52-54]. This may explain some discrepancies reported in the literature upon the prognostic value of intratumoral CD8^+^ T cells in head and neck cancer patients [55]. However, in previous studies we could not demonstrate a relationship between infiltrating CD8^+^ T cells and the clinical outcome, independently of the HPV status of the patients [30]. Other groups claim that the presence of intratumoral CD8^+^ T cell is more predictive of the clinical outcome, when considering the CD4/CD8 [46] or the CD8/CCR4^+^regulatory T cells (Treg) ratios [56,57]. The location of the lymphocytic infiltrates within the tumor (tumor nest or stroma) could also explain the discrepant significance of the intratumoral CD8^+^ T cell presence [56]. In the present work, we accessed the total number of CD8^+^ T cells regardless of their tumor location, due to the practice of non-automated counting and impossibility of accurate discrimination of the CD8^+^ T cells in the various locations.

## Conclusions

We investigated the putative prognosis value of two molecules involved in tumor cell resistance to CD8^+^ T cell-mediated lysis, EPHRIN-A1 and SCINDERIN. Overall, our results show that molecules mediating tumor resistance to CD8^+^ T cells could not be used as reliable markers for prognostic evaluation in head and neck cancer patients. Moreover, in contrast to other tumor types, intratumoral infiltration of CD8^+^ T cells was not a compatible clinical biomarker in head and neck tumors. The prognosis value of these different factors could be clarified by additional analyses of tumor cell phenotype, the profile of their microenvironment or specific features of the tumor cells.

## Competing interests

The authors declare that they have not competing interests.

## Author's contributions

MH, CB, PV, AL, ET analyzed and interpreted the data. CB, MOD, HR, EdG, SO, SH collected data. FD and VM performed immunohistochemistry. MH, CB, ET and SC drafted the manuscript. CB, ET and SC contributed to the conception and design of the study. All authors read and approved the final manuscript.

## Pre-publication history

The pre-publication history for this paper can be accessed here:

http://www.biomedcentral.com/1471-2407/13/592/prepub
